# Antimicrobial activity, chemical composition and mechanism of action of Chinese chive (*Allium tuberosum* Rottler) extracts

**DOI:** 10.3389/fmicb.2022.1028627

**Published:** 2022-11-01

**Authors:** Cun Chen, Jing Cai, Ying-hong Ren, Yue Xu, Hong-ling Liu, Yu-yang Zhao, Xing-fu Chen, Zhi-bin Liu

**Affiliations:** ^1^College of Agronomy, Sichuan Agricultural University, Chengdu, Sichuan, China; ^2^Sichuan Provincial Key Laboratory for Development and Utilization of Characteristic Horticultural Biological Resources, College of Chemistry and Life Science, Chengdu Normal University, Chengdu, Sichuan, China; ^3^West China School of Pharmacy, Sichuan University, Chengdu, Sichuan, China; ^4^Key Laboratory of Bio-Resource and Eco-Environment of Ministry of Education, College of Life Sciences, Sichuan University, Chengdu, Sichuan, China

**Keywords:** Chinese chive (*Allium tuberosum* Rottler), antimicrobial activity, HPLC, GC–MS, 2-amino-5-methylbenzoic acid

## Abstract

Chinese chive (*Allium tuberosum* Rottler) is a popular food from *Allium* species in East and Southeast Asia. Most *Allium* species possess characteristic aromas and have antimicrobial activity. In this study, the antimicrobial activities of root, leaf, and scape extracts of Chinese chive at different pH levels (3.0, 5.0, 7.0, 9.0, and 10.7) were compared. The most pronounced activity was produced by the scape extract, and the greatest activity was obtained at pH 5.0. HPLC and GC–MS analysis showed that the major active ingredient was 2-amino-5-methylbenzoic acid. The mechanism of action of Chinese chive scape extracts may involve the depression or disruption of cell membrane integrity, according to our results of the leakage of electrolytes and protein, as well as scanning electron microscopy and transmission electron microscopy observations.

## Introduction

Microbial plant diseases in the field and during transport, storage, and sale are some of the most important issues facing agriculture worldwide (Toth et al., 2010; Myung et al., 2015). Chemical bactericides are used to control plant diseases, but they are often considered undesirable because of the potential environmental impact of residues (Kaur and Garg, 2014) and the appearance of resistant bacteria (Cao et al., 2021). Therefore, research is focused on efficient and effective alternatives to control fungal and bacterial diseases. Chen T. et al. (2018) conducted *in vitro* experiments with ginger oleoresin to confirm their use in controlling rotten disease in harvested Chinese olive caused by *Pestalotiopsis microspore* (Chen T. et al., 2018). Bordoh et al. (2020) found that extract of dukung anak (medicinal herb) and turmeric had antifungal effect against *Colletotrichum gloeosporioides* (Bordoh et al., 2020). The activity of extracts from oregano, common sage, savory, monarda and clove flower buds were tested *in vitro*, and their antibacterial activity toward *Agrobacterium tumefaciens*, *Erwinia amylovora*, *Pseudomonas syringae* and *Xanthomonas arboricola* were found (Kauna et al., 2021). *Allium* species are widely distributed throughout North America, Europe, North Africa, and Asia, and most of them possess characteristic aromas and are edible (Ozturk et al., 2012; Alam et al., 2022). Chinese chive (*Allium tuberosum* Rottler) is in the same family as onion (*Allium cepa*) and garlic (*Allium sativum*) and it is a very popular food in East Asia and Southeast Asia. People love the fresh taste and unique smell of its leaves and scape. It is a perennial plant that has stems, leaves, and inflorescences which are edible and have antimicrobial and antioxidant activities (Ibrahim et al., 2009), and as a mouthwash to soothe toothaches through their antiseptic effect in Thailand and Indo-China (Jannat et al., 2019). Chinese chive oil has been tested for activity against *Flavobacterium columnare*, which leads to fish disease (Rattanachaikunsopon and Phumkhachorn, 2009). Both the leaf and root leachates and volatiles of Chinese chive were verified to have antifungal activity against *Fusarium oxysporum* f. sp. *cubense* race 4 (FOC), which cause Panama disease of bananas in South China (Hui et al., 2013). Six plants from the Alliaceae family were made into essential oils, and the Chinese chive and onion oils were found to have the highest antibacterial activity on five bacteria, *Staphylococcus aureus*, *Listeria monocytogenes*, *Salmonella Typhimurium*, *Escherichia coli and Campylobacter jejuni* (Dima et al., 2014).

Most studies have focused on essential oils obtained by hydrodistillation of the leaves, bulbs, roots, and seeds of Chinese chive (Hu et al., 2013; Dima et al., 2014; El-Sayed et al., 2017b), but certain volatile components can be degraded by hydrodistillation (Swargiary et al., 2020). Chekki et al. (2014) found that ethanolic extract of garlic had higher anti-bacterial activity than essential oils obtained by hydrodistillation (Chekki et al., 2014), and some researchers have obtained extracts from Chinese chive using organic reagents such as ethanol, methanol, and diethyl ether (Yabuki et al., 2010; Fang et al., 2015; Jang and Yong, 2021), but this is time-consuming and expensive. Previous studies have shown there are different matters with different pH extracts. At higher pH and temperature, extracts of the brown alga *Macrocystis pyrifera* have better activity in promoting the growth of tomato and root of bean (Briceño-Domínguez et al., 2014). Wu et al. (2018) explored changes in the anthocyanin content of Roselle at different pH levels, and found that anthocyanins degraded quickly in low-acid environment (Wu et al., 2018). Chen T. et al. (2018) compared the antimicrobial activity constituents in garlic under different pH values, the results showed that acidic extracts had a significant inhibitory effect, while the alkaline extracts had almost no effect, which has come to our attention (Chen C. et al., 2018).

Studies have shown that extracts from different plant parts have different antimicrobial effects. It was reported that extracts of leaves, outer culm, inner culm, branches, rhizomes, knots, and roots of bamboo have different chemical compositions and antibacterial activities (Akinobu et al., 2014). Raudsepp et al. (2018) compared the antibacterial and antioxidative activities of different parts of blackcurrant (*Ribes nigrum*), garden rhubarb (*Rheum rhaponticum*), chokeberry (*Aronia melanocarpa* (Michx.) Elliott), and blue honeysuckle (*Lonicera caerulea* var. *edulis*) and found that the combination of dark rhubarb roots and black chokeberry berries have substantial antibacterial and antioxidative effects (Raudsepp et al., 2018). The antibacterial effect was compared between bark and leaf extracts of *Eucalyptus camaldulen*sis, and found that crude aqueous leaf extracts show lower activity against *Propionobacterium acnes* than bark (Sabo and Knezevic, 2019). The purpose of our study was first to compare the antimicrobial properties of extracts from the roots, leaves, and scape of Chinese chive obtained at different pH values. Subsequently, the active ingredient with the highest antimicrobial activity was separated and concentrated by high-performance liquid chromatography (HPLC), and then determined by gas chromatography–mass spectrometry (GC–MS) and standard. Finally, the possible mechanism of action for the antimicrobial activity was explored by permeability analysis, cell-membrane integrity assays, scanning electron microscopy (SEM), and transmission electron microscopy (TEM).

## Materials and methods

### Chinese chives extracts preparation

Fresh Chinese chives were purchased from the local market in Chengdu. Data used in this study have been archived in Garden and Botanic (2019). An extraction buffer was prepared as 50 mM Tris HCl solutions in water at different pH values (3.0, 5.0, 7.0, 9.0, and 10.7). The roots, leaves, and scape of Chinese chives (Supplementary Figure S1) were rinsed with water, wiped with paper, and crushed in a mortar. Twenty grams of crushed roots, leaves, or scape was added to 40 ml of water at different pH values for 4 h at 4°C. The mixtures were centrifuged at 8,586 × *g* for 20 min, and the supernatants were filtered through 0.22 μm membrane filters. Finally, each 10 ml sample was obtained. The analytical samples for each pH value were prepared to give three replicates for each.

### Microbial strains and culture

Bacterial strains were *Pectobacterium carotovorumum* (DSM 14774) and *Pseudomonas syringae* (DSM 1241). Fungal strains were *Fusarium proliferatum* (DSM 764) and *Alternaria brassicicola* (DSM 62008). All microbial strains were obtained from Beina Chuanglian Biological Research Institute (Beijing, China).

Bacteria were grown in nutrient broth (NB) on an orbital shaker at 28°C, and fungi were cultivated on potato dextrose agar (PDA) plates at 28°C.

### Antimicrobial tests

Antimicrobial tests used Oxford cup assays (Shang et al., 2014) with some modifications. Bacteria were grown to an OD_600_ of 0.6 in NB agar at 200 rpm and 28°C in an orbital shaker. The NB agar medium was cooled to 50°C after autoclaving, and then agar (100 ml) was mixed with bacterial solution (1 ml) and added to the plates. Five 6 mm diameter Oxford cups were placed at equal distances on the agar surface. Chinese chive extracts (50 μl) at different pH values were added to each cup with a micropipette.

Fungal spores were washed with sterile water to 2 × 10^7^ ml^−1^ in a hemocytometer and were stored at 4°C until use (Yin and Cheng, 1998). Antifungal tests were similar to the antibacterial tests, 100 ml PDA agar was mixed with 1 ml spores solution and added to the plates, 50 μl Chinese chive extracts were added to each cup with a micropipette.

The size of the inhibition zones of bacteria were observed after 24 h at 28°C, while the fungi were cultured for 2–4 days. Each treatment was repeated in triplicate.

### High-performance liquid chromatography

The extracts were diluted with distilled water to 2% solutions, and an Alltima C18 column (250 × 4.5 mm, 5 μm) was injected with 20 μl of the diluted extracts and monitored at 260 nm and 25°C. The mobile phase was acetonitrile/water solution (20:80, *v*/*v*), introduced at a flow rate of 0.3 ml/min. The peaks were collected with an Agilent Zorbax Eclipse XDB-C18 (250 × 9.4 mm, 5 μm). Five kg of samples were used to obtain 2.5 l extract. The extract was evaporated *in vacuo* to afford 100 ml concentrated extract. Then, the concentrated extract was subjected to semipreparative HPLC. After 5 times separation and concentration, the compounds were finally obtained. The analyses were performed on a Shimadzu LC-20A liquid chromatograph with a diode array detector (CBM20A, Shimadzu, Tokyo, Japan).

### Gas chromatography–mass spectrometry

The compounds were analyzed by GC–MS (GC/MS-QP2010, Shimadzu, Kyoto, Japan) using an Rtx-Wax capillary column (30 m, 0.25 mm, and 0.25 μm). The ionization voltage was 70 eV, and the carrier gas was helium flowing at 1 ml/min. The oven temperature program was 60°C for 5 min, which was then increased to 280°C at a rate of 10°C/min while the injection port temperature was maintained at 220°C. The MS data libraries NIST05.LIB and NIST05s.LIB were used to analyze the spectrum and identify the compounds.

### Cell membrane permeability

#### Fungi (*Fusarium proliferatum*)

Mycelial samples were prepared according to the method of Medina-Romero et al. (2017) with some modifications. *F. proliferatum* was cultivated on PDA plates for 6 days, and the plates were washed with 100 ml of double-distilled sterile water to wash away the spores. Every 100 mg of moist mycelium was then mixed with 10 ml of double-distilled sterile water, and 500 μl of Chinese chive scape extracts (pH 3.0, 5.0, 7.0, 9.0, and 10.7), 50 mM Tris HCl solutions at pH 5.0 (negative control, N) or allicin (positive control, P) was added. Mycelium suspensions were shaken at 120 rpm and 28°C in an orbital shaker for 2 to 12 h.

#### Bacteria (*Pectobacterium carotovorum*)

Bacterial samples were grown to OD_600_ ≈ 0.6 in NB, and each 10 ml culture was treated with 500 μl of Chinese chive scape extracts, 50 mM Tris HCl solutions at pH 5.0 (negative control, N) or allicin (positive control, P), then incubated for 2–12 h.

The electrical conductivity of samples was measured using a conductivity meter (DDS-307, SPSIC-Rex Instrument Factory, China).

### Integrity of the cell membrane

Fungal (*F. proliferatum*) mycelium and bacterial (*P. carotovorum*) samples were prepared in the same manner as the electrical conductivity experiments, with the bacteria incubated for 4–12 h and the fungi incubated for 4–16 h. The microbial culture media treated with Chinese chive scape extracts were centrifuged for 15 min at 3,354 × *g*, and the supernatants were obtained. Each 200 μl supernatant was mixed with 800 μl of Coomassie Brilliant Blue G-250 for 10 min, and then the mixtures were analyzed on a T6-spectrophotometer (Beijing Purkinje General Instrument Co., Ltd., Beijing, China) at a wavelength of 595 nm. The protein leakage could reflect the integrity of the microbial cell membrane.

### Scanning electron microscopy

The *P. carotovorum* cells were grown in NB agar at 200 rpm and 28°C in an orbital shaker overnight. Then 10 ml bacteria culture samples were treated with 800 μl of 50 mM Tris HCl solutions at pH 5.0 (control), 400 μl of 50 mM Tris HCl solutions at pH 5.0, 400 μl of Chinese chive scape extracts (pH 5.0), and 800 μl Chinese chive scape extracts (pH 5.0). After 12 h of incubation, the mixtures were centrifuged at 3,354 × *g* for 15 min. The precipitated cells were washed with phosphate-buffered saline (PBS, 0.1 mol/l, pH 7.4) and fixed with 2.5% (w/v) glutaraldehyde at 4°C for 12 h. Subsequently, the samples were rinsed with PBS for 1 min and dehydrated in a graded series (30, 50, 70, 80, and 90% ethanol) for 15 min, then in 100% ethanol for 15 min twice, respectively. The samples were dried in critical-point liquid CO_2_ and coated with gold by cathodic spraying. A scanning electron microscope (JSM-750 0F, JEOL Ltd., Tokyo, Japan) was used to examine the final samples.

### Transmission electron microscopy

Transmission electron microscopy (TEM) procedure was carried out using Lesley’s method (Lesley and Jan Marc, 2007) with some modifications. *F. proliferatum* was cultivated on PDA plates with added treatment fluid at 28°C for 72 h (A: 200 μl 50 mM Tris HCl solutions at pH 5.0; B: 100 μl 50 mM Tris HCl solutions at pH 5.0 + 100 μl Chinese chive scape extracts, pH 5.0; C: 200 μl Chinese chive scape extracts, pH 5.0). Segments of *F. proliferatum* grown on the PDA plates (1 mm × 1 mm dimensions) were cut and filled with 2.5% (v/v) glutaraldehyde for 2 h and then rinsed three times with phosphate buffer (PB, 0.1 mol/l, pH 7.4) for 15 min each. Next, the samples were fixed with 1% citric acid at room temperature for 5 h and washed with PB three times (15 min each). After the samples were rinsed, they were immersed in an ethanol series (1 h each in 30, 50, 70, 80, 90, and 95% ethanol; twice for 1 h in 100% ethanol) and subsequently immersed in an ethanol/acetone series (3,1, 1:1, and 1:3, *v*/*v*, 30 min each), and in acetone for 1 h. The samples were then embedded with an acetone/Epon812 series (3, 12, 4 h; 1:1, 12 h; 1:3, 4 h). After the samples were embedded in Epon812 8 h, they were inserted into the embedding plate in an oven at 37°C for 12 h and at 60°C for 48 h. The embedded samples were sliced into 60–80 nm ultrathin slices with an ultramicrotome (EM UC7, Leica, Austria), stained with saturated uranyl acetate solution (2%) in alcohol for 15 min, and then stained with lead citrate (2%) for 15 min. After overnight drying, the final samples were observed by TEM (Hitachi-HT7700, Japan).

## Results

### *In vitro* activity of different Chinese chive extracts against plant pathogenic microorganisms

**Figure 1:**
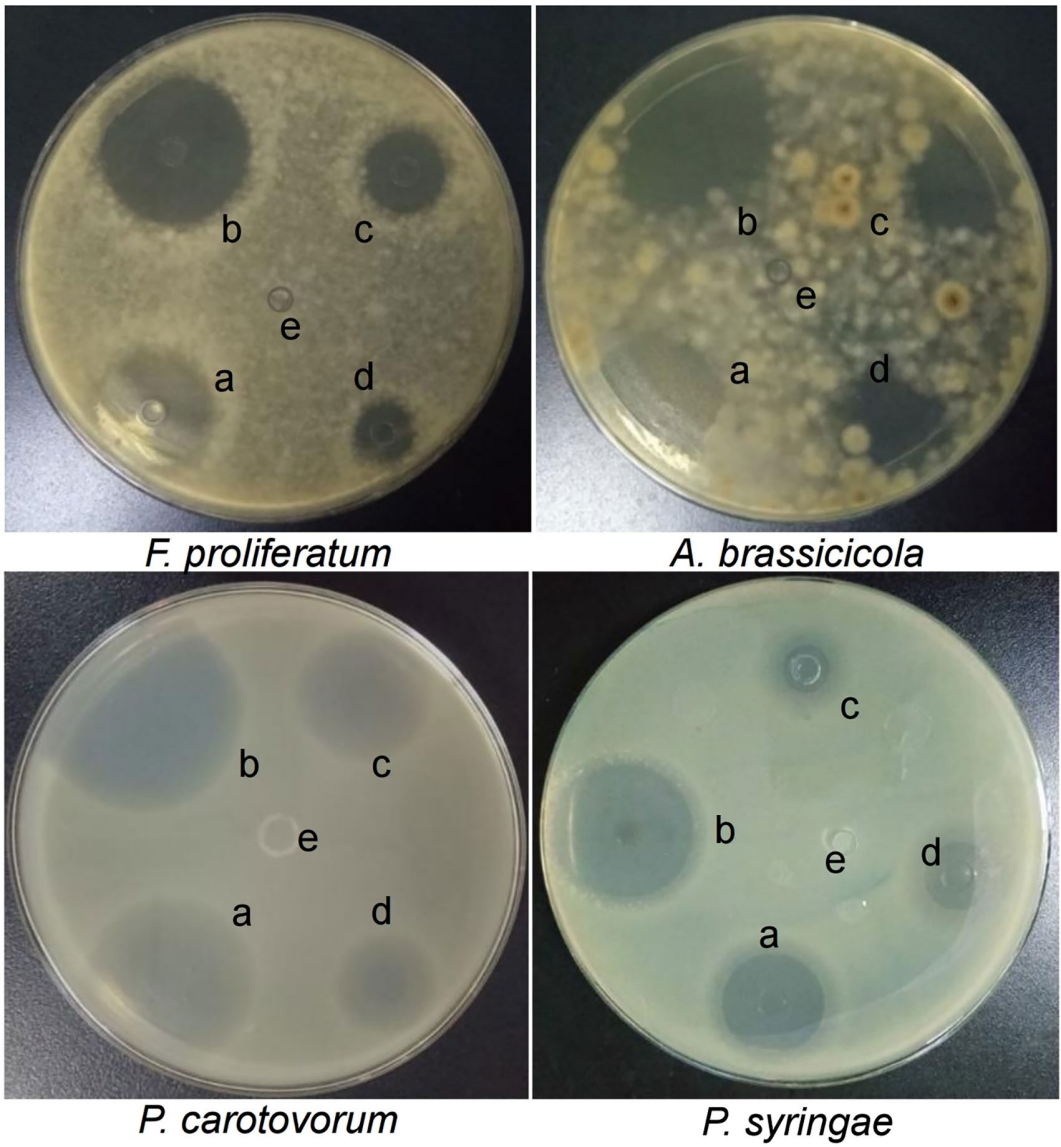
Zones of inhibition caused by Chinese chive scape extracts on seeded agar plates. a: pH 3.0; b: pH 5.0; c: pH 7.0; d: pH 9.0; e: pH 10.7.

Bacterial growth (*P. carotovorum* and *P. syringae*) and fungal growth (*F. proliferatum* and *A. brassicicola*) were inhibited by extracts of different parts of Chinese chive on seeded agar plates. As shown by the results in Supplementary Table S1, the scape of Chinese chive extracts had the most significant activity. The zones of inhibition with extracts of scape at different pH was shown in Figure 1. The pH 5.0 extracts had the highest activity against the microorganisms, followed by extracts at pH 3.0, pH 7.0, and pH 9.0; the extracts at pH 10.7 had no inhibitory effect, and 50 mM Tris HCl solutions at different pH had no activity (Supplementary Figure S2).

### High-performance liquid chromatography analysis of Chinese chive scape extracts

**Figure 2:**
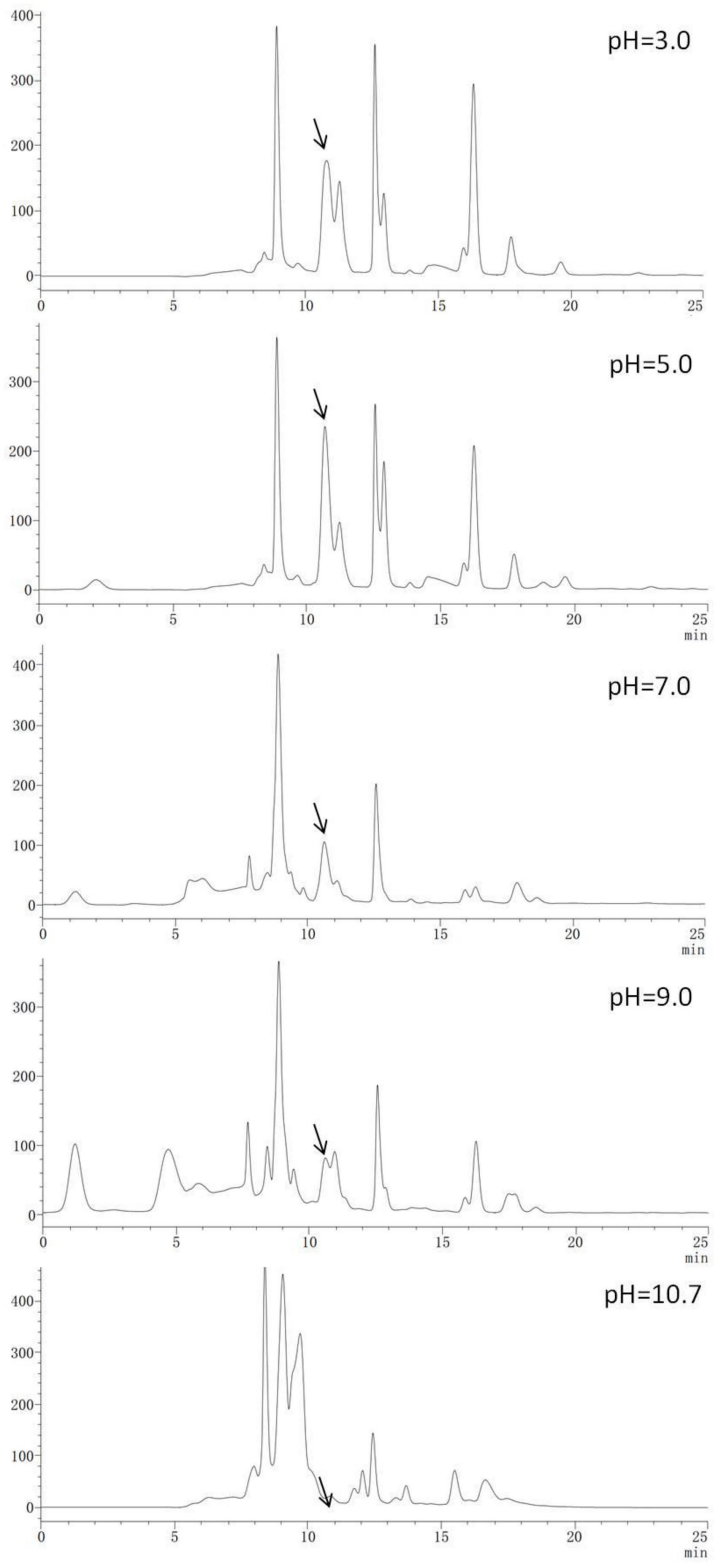
High-performance liquid chromatography–UV_260nm_ chromatograms of Chinese chive scape extracts.

HPLC-UV_260nm_ chromatograms for Chinese chive scape extracts are shown in Figure 2. The absorption peak appearing at about 10.6 min (arrow) and 12.5 min was much stronger in the pH 5.0 and 3.0 extracts, followed pH 7.0, and 9.0, in descending order. There was no peak in this position for the pH 10.7 extracts. Interestingly, the height of the peak at 10.6 and 12.5 min was positively correlated with the size of the inhibition zone. Therefore, we speculated that the 10.6 and 12.5 min peak had an active substance, and we collected and concentrated the two peaks and performed an antimicrobial test (Supplementary Figure S3), but found that only the 10.6 min peak had activity, it showed this 10.6 peak had the active substance.

### Gas chromatography–mass spectrometry analysis of active constituents

**Figure 3:**
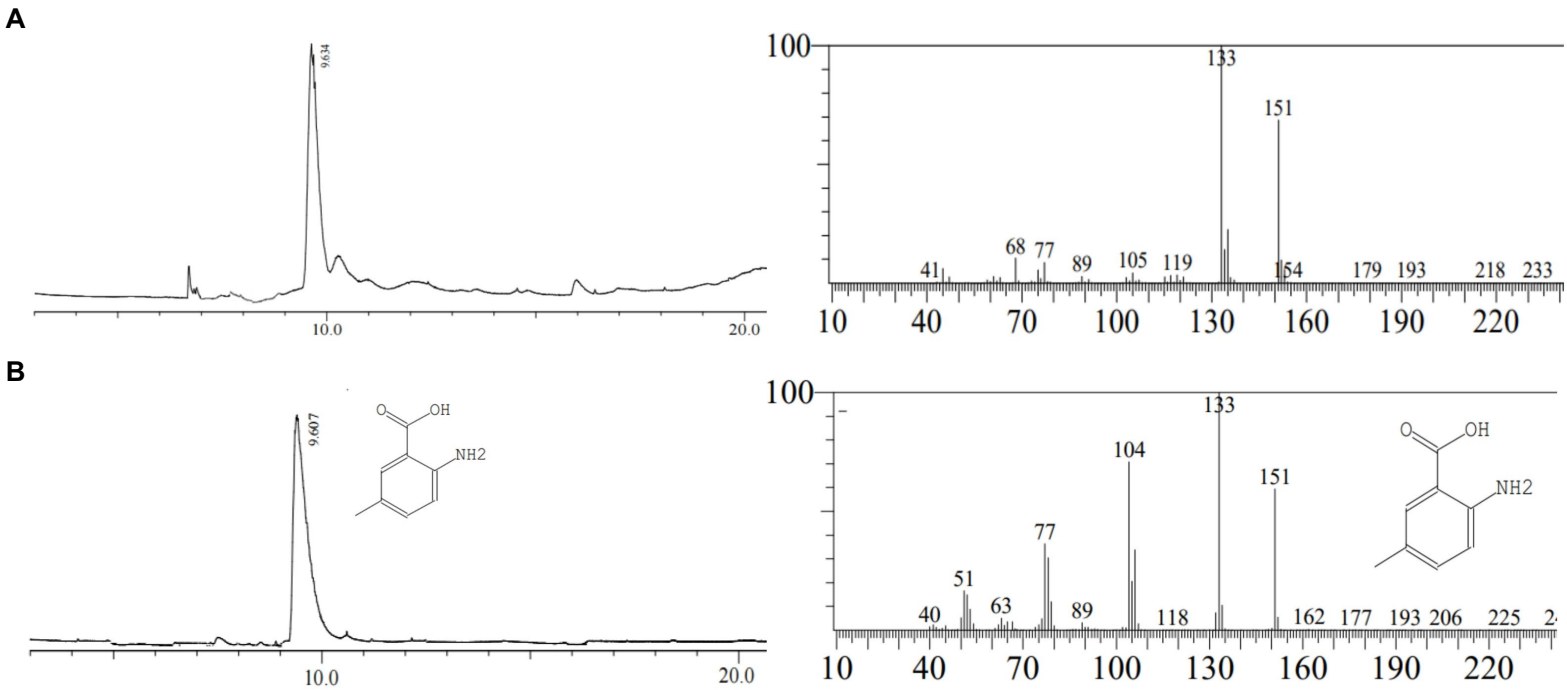
Gas chromatography–mass spectrometry analysis. **(A)**: the peak of 10.6 min in HPLC; **(B)**: 2-amino-5-methylbenzoic acid (standard).

In this study, the 10.6 min peak (Figure 2) isolated and purified from Chinese chive scape extracts was analyzed by GC–MS. The results identify 2-amino-5-methylbenzoic acid (CAS: 2941-78-8) as the major compounds at levels of 86.10% (Figure 3A). Further verification of the antimicrobial activity of the substance revealed that 2-amino-5-methylbenzoic acid (standard compound) has antimicrobial activity (Supplementary Figure S4), like allicin (Supplementary Figure S5).

**Figure 4:**
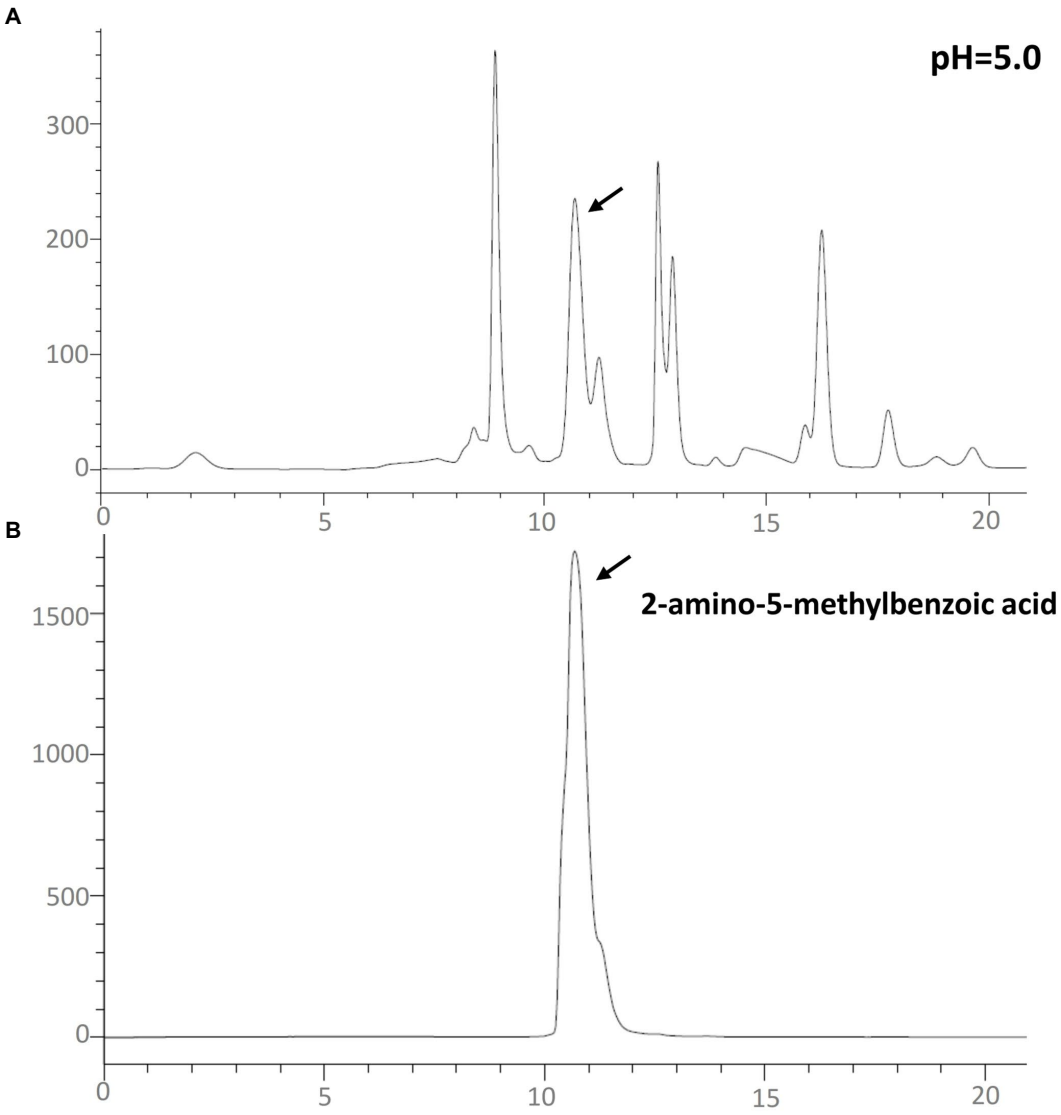
High-performance liquid chromatography–UV_260nm_ chromatograms. **(A)** Chinese chive pH 5.0 scape extracts; **(B)** the standard of 2-amino-5-methylbenzoic acid.

In order to confirm that the compound is 2-amino-5-methylbenzoic acid, the Chinese chive scape extracts (pH 5.0) and 2-amino-5-methylbenzoic acid (standard) were tested by GC–MS and HPLC. The mass spectra of 2-amino-5-methylbenzoic acid (standard compound) was shown in Figure 3B, and the time of the 10.6 min peak and 2-amino-5-methylbenzoic acid (standard compound) in HPLC was consistent (Figure 4).

### Cell membrane permeability of microorganisms

**Figure 5:**
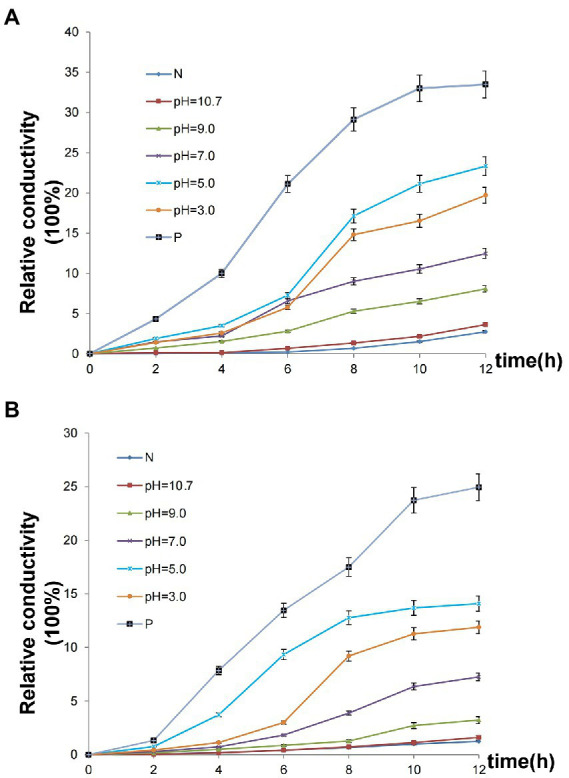
Effect of Chinese chive scape extracts on the membrane permeability. Values are mean ± SD (n = 3). **(A)** *P. carotovorum*; **(B)** *F. proliferatum*. N: negative control, 50 mM Tris HCl solutions at pH 5.0; P: positive control, allicin.

An assay of cell membrane permeability was used to study the effect of the Chinese chive scape extracts on *P. carotovorum* (Figure 5A) and *F. proliferatum* (Figure 5B) cell surfaces. The relative conductivity of both the negative control and pH 10.7 extracts changed little. In contrast to the negative control, the extracts at pH 3.0, 5.0, 7.0, 9.0 and positive control had varying degrees of increase after 12 h. Especially the extracts at pH 5.0, a large increase was observed at 4–6 h, followed pH 3.0, a significant increase was observed at 6–8 h.

### Leakage of cellular contents of microorganisms

**Figure 6:**
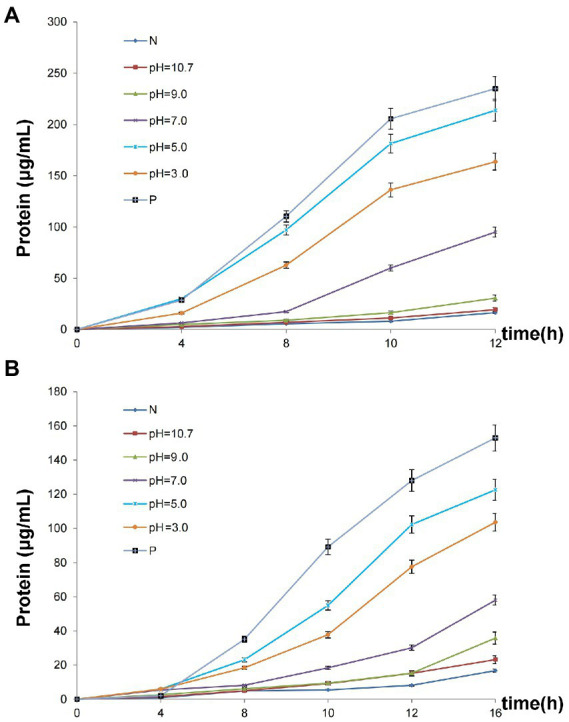
Leakage of protein. Values are mean ± SD (*n* = 3). **(A)** *P. carotovorum*; **(B)** *F. proliferatum*. N: negative control, 50 mM Tris HCl solutions at pH 5.0; P: positive control, allicin.

Chinese chive scape extracts elevated protein leakage through the plasma membrane of *P. carotovorum* (Figure 6A) and *F. proliferatum* (Figure 6B). As shown in Figure 6A, leakage of proteins from the negative control, pH 10.7 and pH 9.0 bacterial was almost not observed protein leakage. The pH 7.0 extracts caused visible protein leakage after 10 h, while the positive control, pH 5.0 and 3.0 extracts caused extensive protein leakage after 8 h.

Fungal leakage appeared slower than bacteria, so time was extended to 16 h. There was little change in the negative control, pH 10.7, and pH 9.0 treatments during the first 12 h, and then a slight increase was observed at 16 h. The positive control and pH 5.0 extracts led to the most obvious protein leakage as bacteria, a significant increase was observed at 8 h, followed by pH 3.0, and then pH 7.0 extracts (Figure 6B).

### Scanning electron microscopy analysis of bacteria

**Figure 7:**
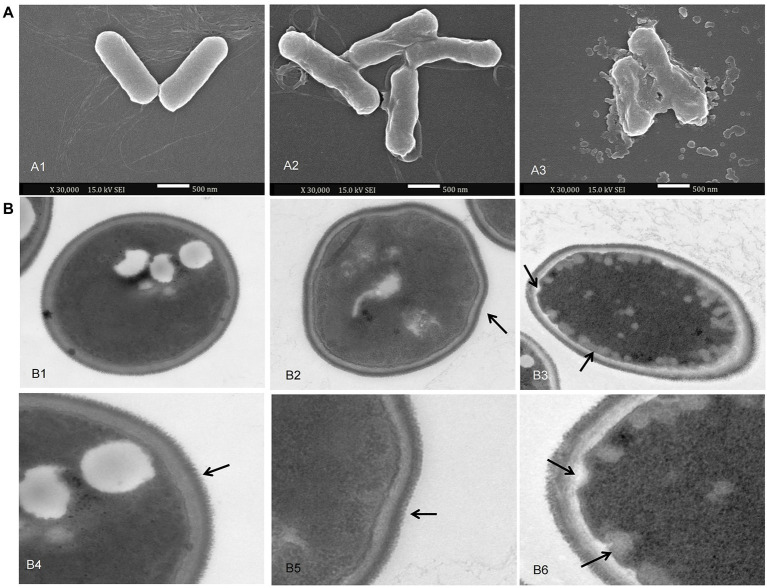
scanning electron microscopy (SEM) and transmission electron microscopy (TEM) images. **(A)**: SEM images of *P. carotovorum*. A1: addition of 800 μl of 50 mM Tris HCl solutions at pH 5.0 per 10 ml culture; A2: addition of 400 μl of 50 mM Tris HCl solutions at pH 5.0 + 400 μl of pH 5.0 scape extracts per 10 ml culture; A3: addition of 800 μl of pH 5.0 scape extracts per 10 ml culture. **(B)**: TEM images of *F. proliferatum*. B1, B4: addition of 200 μl of 50 mM Tris HCl solutions at pH 5.0; B2, B5: addition of 100 μl of 50 mM Tris HCl solutions at pH 5.0 + 100 μl of pH 5.0 scape extracts; B3, B6: addition of 200 μl of pH 5.0 scape extracts.

To explain the changes in relative conductivity and protein leakage, morphological changes in the appearance of the bacteria (*P. carotovorum*) cells were observed by SEM. *P. carotovorum* were treated with 50 mM Tris HCl solutions at pH 5.0 (control) and Chinese chive scape extracts at pH 5.0 for 12 h. The SEM images in Figure 7A show the surface morphology and physical changes of the samples. The bacteria in the control group (Figure 7A1) were complete and regular, while bacteria treated with pH 5.0 extracts (Figures 7A2,A3) became deformed and broken and adhered to each other, with the changes more evident as extract concentration increased. SEM observations confirmed the damage to the structural integrity of the tested bacteria, which supported the results of the permeability and integrity tests on the bacteria cells.

### Transmission electron microscopy analysis of fungi

The ultrastructure of *F. proliferatum* was investigated by TEM to further explain the inhibitory mechanism of Chinese chive scape extracts. *F. proliferatum* grown without extracts showed a normal, intact cell structure, and a thick cell wall (Figure 7B1), and the filiform was regular and thick as the arrow referred (Figure 7B4). On the contrary, the pH 5.0 extract induced depressed cellular surfaces, and plenty of lipid globules beside the cell membrane (Figures 7B2,B3,B5,b6), as the arrow referred. Furthermore, the filiform became irregular (Figures 7B5,B6).

## Discussion

In this work, the extracts of roots, leaves, and scape of Chinese chive at different pH values were tested as antimicrobial agents against two bacterial (*P. carotovorum* and *P*. *syringae*) and two fungal (*F*. *proliferatum* and *A*. *brassicicola*) strains. The results show that the scape extracts had the most significant antimicrobial effects, especially at pH 5.0. HPLC showed that the peak at about 10.6 min was consistent with the inhibition zone experiment. The microbial inhibition test and GC–MS analysis results after collection and concentration of this peak suggest that 2-amino-5-methylbenzoic acid was the inhibitor of microorganisms in Chinese chive.

Traditionally, *Allium* species have been considered to have antimicrobial activity. Most *Allium* species possess characteristic aromas and are edible. Garlic (*A. sativum*) has been used for centuries as a spice with important medicinal properties and has been found to be active against fungi and bacteria. Research has shown that 28 garlic cultivar extracts inhibited four different phytopathogenic fungi (Sikandar et al., 2016). Bacterial spot disease of tomato and pepper wee inhibited by aqueous garlic (Mirik and Aysan, 2005). Onion (*A. cepa*) is also well-known as a flavoring agent, the essential oils of which are potent inhibitors of microbial growth (Llanaruizcabello et al., 2015). Ethanol, methanol, acetone, and ethyl acetate extracts of onion were evaluated for their antibacterial activity against 15 bacterial strains, the results showed that the ethyl acetate extract had higher bactericidal effect due to the presence of high concentration of phenolic compounds (Mahomoodally et al., 2018). Many studies have shown that organic sulfur compounds provide the antimicrobial effect of *Allium* species, such as such as diallyl trisulfide (DTS), diallyl disulfide (DDS) in garlic (Oommen et al., 2004; El-Sayed et al., 2017a). Chinese chive share a common sulfur biochemistry as garlic and onion. *Allium* also contains several aromatic bioactive compounds, and some studies have demonstrated antimicrobial activity from these, such as ferulic acid and caffeic acid which have been detected in garlic, onion, and three-cornered leek (*A. triquetrum*; Prakash et al., 2007; Beato et al., 2011; Rabah et al., 2020). And the change of substituents on the benzene ring have been reported to be an important factor for inducing antimicrobial activity (Uyanik et al., 2010; Reddy et al., 2018). 2-Amino-5-methylbenzoic acid is also an aromatic compound, and it has also been found in the extracts of different varieties of garlic, but its antibacterial activity has not attracted attention (Chen et al., 2019).

2-Amino-5-methylbenzoic acid is an important material that can synthesize drugs. It was mixed with chloroformate in pyridine to form cetilistat, which is a pancreatic lipase inhibitor for the treatment of obesity (Kopelman et al., 2007; Ding et al., 2015). Treatment of 2-amino-5-methylbenzoic acid with butyl isothiocyanate resulted in 2-thioxoquinazolin-4-one, which was used *in vitro* against the HeLa and MDA-MB231 cancer cell lines, and it exhibited significant *in vitro* cytotoxicity against both the cell lines (Abuelizz et al., 2017). 2-Amino-5-methylbenzoic acid could also produce 5-methyl-2-[(phenylsulfonyl)amino]benzoic acid as a potent anti-prostate cancer agent (Purushottamachar et al., 2008). A series of artemisinin-4-(arylamino)quinazolines, which are designed and synthesized from the starting material 2-amino-5-methylbenzoic acid, showed potent *in vitro* cytotoxic activity against colorectal cancer (CRC) HCT116 and WM-266-4 cell lines (Wang et al., 2019). It was found that 2-amino-5-methylbenzoic acid could produce 2-[(benzyloxycarbonyl) amino]-5-methylbenzoic acid, which had the ability to inhibit serine protease (Gütschow et al., 1998). It was also reported that 2-amino-5-methylbenzoic acid could form quinazolinone derivatives that are human acrosin (a serine protease) inhibitors (Weiwei et al., 2013). Serine proteases are widely found in bacteria and fungi (Lizbeth, 2002), and they have many beneficial effects on the microorganism, including degradation of misfolded proteins in the periplasm (Pallen and Wren, 2010), digestion of abnormal proteins within the periplasmic space (Tim et al., 2011), and enabling of protein–protein interactions (Backert et al., 2013). Previous studies showed that suppression of serine protease activity could sufficiently and effectively kill bacteria (Joanna et al., 2013; Tegtmeyer et al., 2016).

The antimicrobial activity of 2-amino-5-methylbenzoic acid have not been reported. In this study, 2-amino-5-methylbenzoic acid from the Chinese chive extracts may killed the test bacterial and fungal strains by depress or disrupt the cell walls and plasma membranes. Cell walls and membranes provide conditions for the selective permeation of small ions such as K^+^ and Na^+^ (Wen-Rui et al., 2014). The observed changes at high relative conductivity and protein leakage indicated that the Chinese chive scape extracts could destroy the structural integrity of the cell wall and membranes. The SEM and TEM images showed changes of cells walls and plasma membranes. The physical and morphological changes of bacterial cells were due to the effect of extracts on the permeability and integrity of membrane, result in a bacterial morphological transition from rod-shaped cells to amorphous cells (Tian et al., 2020; Efenberger-Szmechtyk et al., 2021). Li et al. (2016) found that *F. proliferatum* exhibited the disruption of mycelia in the presence of butylated hydroxyanisole, and cellular surfaces depressed (Li et al., 2016), which confirmed our results. These were the same as the result for the changes in membrane permeability and leakage of cellular contents. Therefore, the direct damage of 2-amino-5-methylbenzoic acid from the Chinese chive extracts to the microbial cells walls and membranes may be an antimicrobial mechanism.

## Conclusion

In this study, we compared the antimicrobial properties of root, leaf, and scape extracts of Chinese chive obtained under different pH values, and we found that the scape had the most significant activity. The scape extracts were then used as an antimicrobial agent against both four test bacterial and fungal strains. We conclude that 2-amino-5-methylbenzoic acid is the main antimicrobial substance of extracts of Chinese chive, and the mechanism of action is the direct damage to the microbial cell membrane.

## Data availability statement

The original contributions presented in the study are included in the article/Supplementary material, further inquiries can be directed to the corresponding author.

## Author contributions

CC: methodology, formal analysis, and writing: original draft. JC: methodology and writing: review and editing. Y-hR: writing: review and editing. YX, H-lL, and Y-yZ: formal analysis. X-fC and Z-bL: conceptualization, resources, writing: review and editing, and funding acquisition. All authors contributed to the article and approved the submitted version.

## Funding

This work was financially supported by Sichuan Province Science and Technology Support Program (no. 2021YFN0119 and 2022ZHXC0009), the Program for Innovative Research Team of Chengdu Normal University (no. CSCXTD2020A04), the On-Job Doctoral Fund of Chengdu Normal University (no. ZZBS2020-12), and Discipline Construction Project of Sichuan Agricultural University (no. 03572976).

## Conflict of interest

The authors declare that the research was conducted in the absence of any commercial or financial relationships that could be construed as a potential conflict of interest.

## Publisher’s note

All claims expressed in this article are solely those of the authors and do not necessarily represent those of their affiliated organizations, or those of the publisher, the editors and the reviewers. Any product that may be evaluated in this article, or claim that may be made by its manufacturer, is not guaranteed or endorsed by the publisher.

## Supplementary material

The Supplementary material for this article can be found online at: https://www.frontiersin.org/articles/10.3389/fmicb.2022.1028627/full#supplementary-material

SUPPLEMENTARY FIGURE S1 Chinese chive (Allium tuberosum Rottler). a: roots; b: leaves; c: scape.

SUPPLEMENTARY FIGURE S2 Oxford cup test of 50 mM Tris HCl solutions at different pH. a: pH 3.0; b: pH 5.0; c: pH 7.0; d: pH 9.0.

SUPPLEMENTARY FIGURE S3 The pH 5.0 HPLC-UV peaks appearing at about 10.6 min and 12.5 were collected and concentrated to test the antimicrobial activity. a & b: peak appearing at 10.6 min; c & d: peak appearing at 12.5 min.

SUPPLEMENTARY FIGURE S4 Zones of inhibition caused by 50 μL of 2-amino-5-methylbenzoic acid. a: 6.25 μg/mL; b: 12.5μg/mL; c: 25 μg/mL.

SUPPLEMENTARY FIGURE S5 Zones of inhibition caused by 50 μL of 2-amino-5-methylbenzoic acid and allicin. a: 25 μg/mL of allicin; b: 25 μg/mL of 2-amino-5-methylbenzoic acid; c: 50 mM Tris HCl solutions at pH 5.0.
