# Supplementary figures and images for: Antimicrobial activity, chemical composition and mechanism of action of Chinese chive (Allium tuberosum Rottler) extracts
Source: Front Microbiol. 2022 Nov 1;13:1028627. doi: 10.3389/fmicb.2022.1028627 (PMC9664698; doi:10.3389/fmicb.2022.1028627)

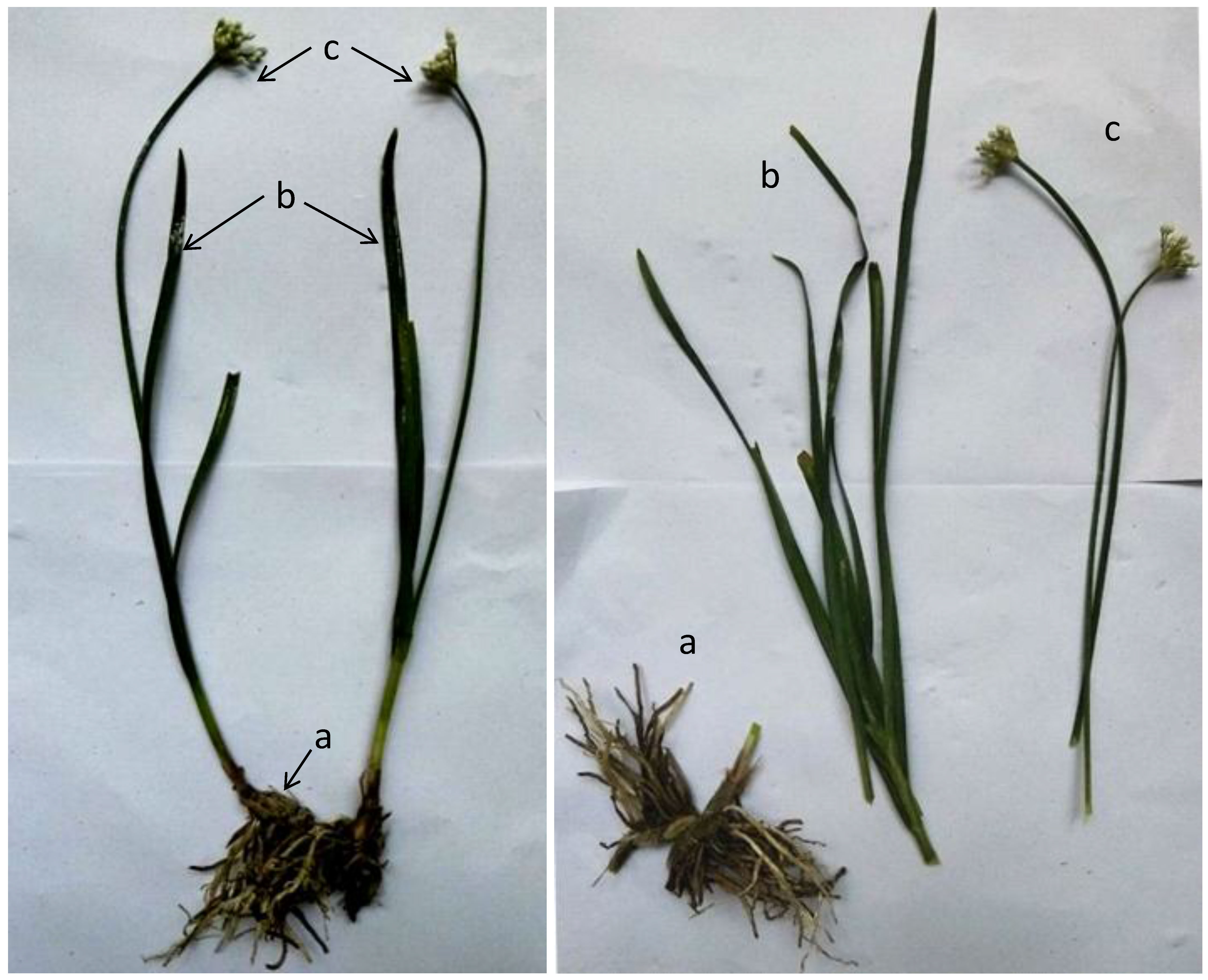

Supplement: SUPPLEMENTARY FIGURE S1 — Chinese chive (Allium tuberosum Rottler). a: roots; b: leaves; c: scape. [file Image_1.JPEG]

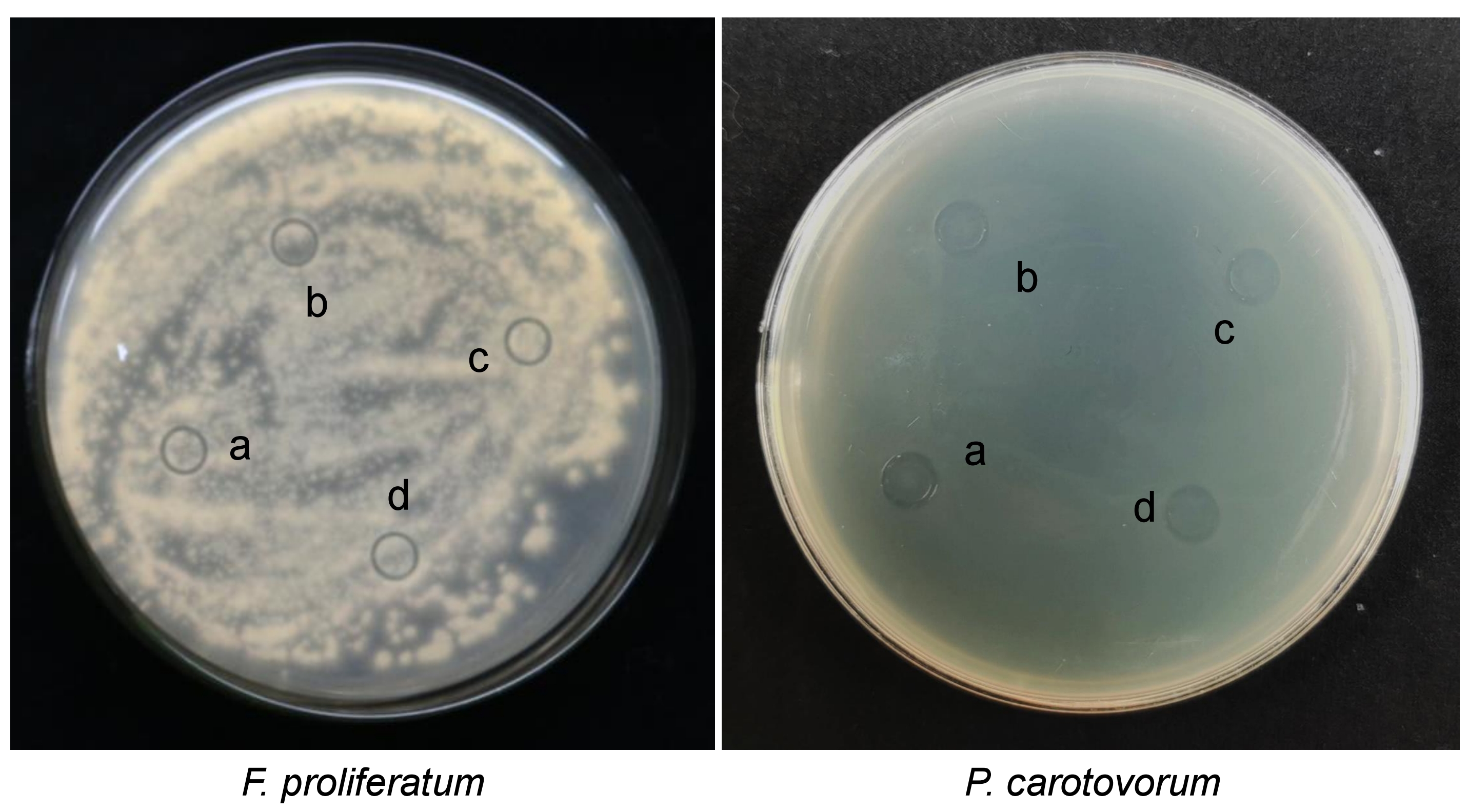

Supplement: SUPPLEMENTARY FIGURE S2 — Oxford cup test of 50 mM Tris HCl solutions at different pH. a: pH 3.0; b: pH 5.0; c: pH 7.0; d: pH 9.0. [file Image_2.JPEG]

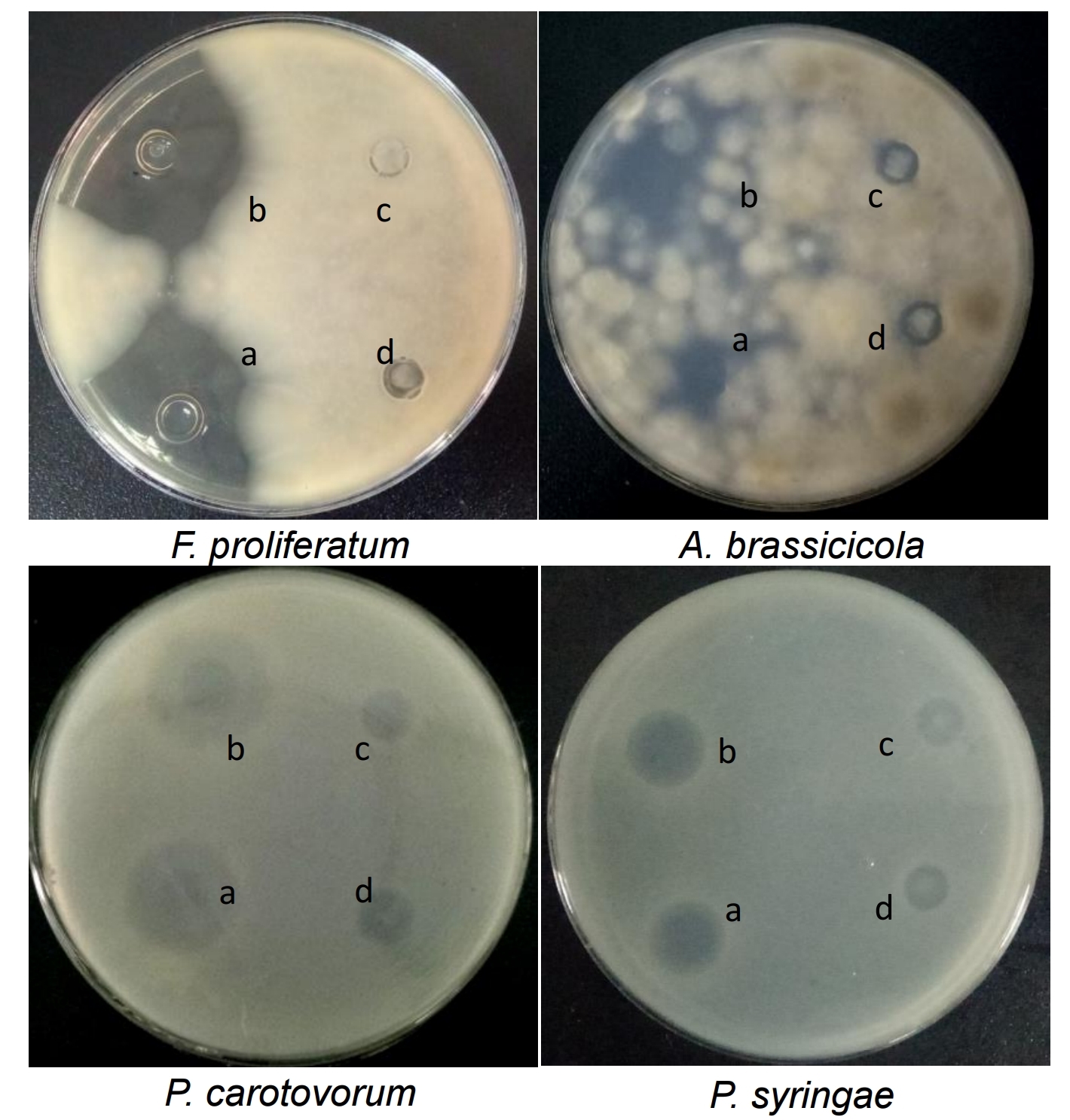

Supplement: SUPPLEMENTARY FIGURE S3 — The pH 5.0 HPLC-UV peaks appearing at about 10.6 min and 12.5 were collected and concentrated to test the antimicrobial activity. a & b: peak appearing at 10.6 min; c & d: peak appearing at 12.5 min. [file Image_3.JPEG]

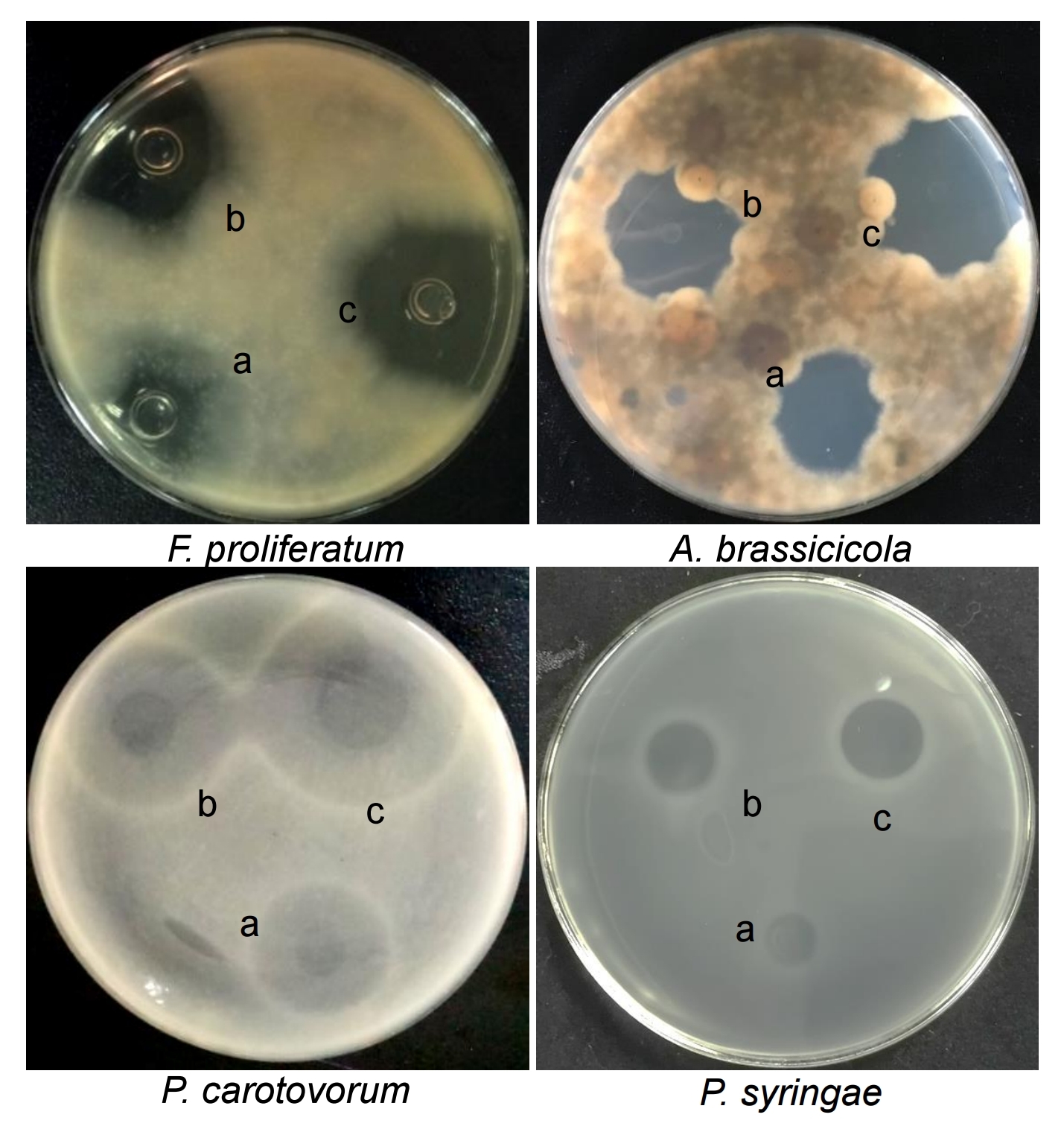

Supplement: SUPPLEMENTARY FIGURE S4 — Zones of inhibition caused by 50 μL of 2-amino-5-methylbenzoic acid. a: 6.25 μg/mL; b: 12.5μg/mL; c: 25 μg/mL. [file Image_4.JPEG]

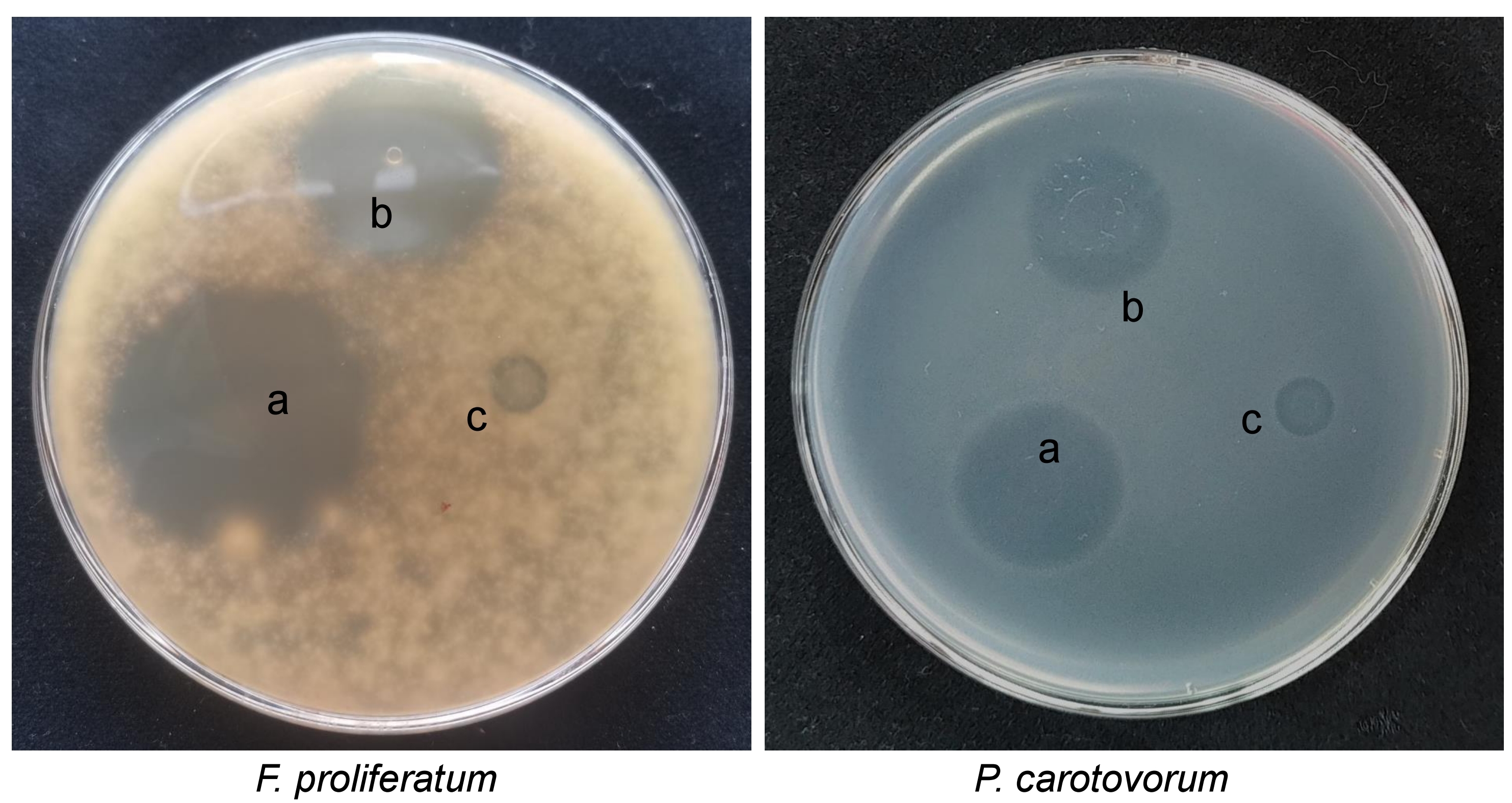

Supplement: SUPPLEMENTARY FIGURE S5 — Zones of inhibition caused by 50 μL of 2-amino-5-methylbenzoic acid and allicin. a: 25 μg/mL of allicin; b: 25 μg/mL of 2-amino-5-methylbenzoic acid; c: 50 mM Tris HCl solutions at pH 5.0. [file Image_5.JPEG]
