# Supplementary material for: Antimicrobial activity, chemical composition and mechanism of action of Chinese chive (Allium tuberosum Rottler) extracts
Source: Front Microbiol. 2022 Nov 1;13:1028627. doi: 10.3389/fmicb.2022.1028627 (PMC9664698; doi:10.3389/fmicb.2022.1028627)
Supplement: Supplementary file 6 [file Table_1.DOCX]

**Appendix B: Antimicrobial test of the roots, leaves, and scapes of Chinese chive extracts with deionized water at different pH levels.**

| **Microorganism** | **pH** | **Zones of inhibition（cm）** | | |
| --- | --- | --- | --- | --- |
|  |  | **Roots** | **Leaves** | **Scapes** |
| ***F. proliferatum*** | pH 3.0 | 1.57 ± 0.21* | 2.64 ± 0.09 | 3.43 ± 0.16 |
|  | pH 5.0 | 2.34 ± 0.11 | 3.71 ± 0.24 | 4.25 ± 0.09 |
|  | pH 7.0 | 1.10 ± 0.05 | 1.60 ± 0.08 | 2.22 ± 0.18 |
|  | pH 9.0 | 0.56 ± 0.06 | 0.97 ± 0.16 | 1.58 ± 0.21 |
|  | pH 10.7 | 0.00 | 0.00 | 0.00 |
|  |  |  |  |  |
| ***A.brassicicola*** | pH 3.0 | 2.30 ± 0.15 | 3.03 ± 0.16 | 3.85 ± 0.09 |
|  | pH 5.0 | 3.17 ± 0.14 | 3.89 ± 0.15 | 5.18 ± 0.14 |
|  | pH 7.0 | 1.30 ± 0.13 | 2.04 ± 0.07 | 3.23 ± 0.09 |
|  | pH 9.0 | 0.59 ± 0.14 | 1.36 ± 0.08 | 2.10 ± 0.16 |
|  | pH 10.7 | 0.00 | 0.00 | 0.00 |
|  |  |  |  |  |
| ***E. carotovora*** | pH 3.0 | 1.32 ± 0.09 | 2.20 ± 0.16 | 2.59 ± 0.16 |
|  | pH 5.0 | 2.87 ± 0.16 | 4.19 ± 0.17 | 5.39 ± 0.37 |
|  | pH 7.0 | 1.13 ± 0.10 | 1.75 ± 0.12 | 3.51 ± 0.25 |
|  | pH 9.0 | 0.57 ± 0.04 | 0.85 ± 0.13 | 2.10 ± 0.16 |
|  | pH 10.7 | 0.00 | 0.00 | 0.00 |
|  |  |  |  |  |
| ***P.syringae*** | pH 3.0 | 1.23 ± 0.19 | 1.94 ± 0.14 | 2.64 ± 0.11 |
|  | pH 5.0 | 2.04 ± 0.20 | 3.69 ± 0.14 | 4.32 ± 0.24 |
|  | pH 7.0 | 1.01 ± 0.08 | 1.50 ± 0.20 | 1.87 ± 0.48 |
|  | pH 9.0 | 0.61 ± 0.04 | 0.79 ± 0.07 | 1.36 ± 0.38 |
|  | pH 10.7 | 0.00 | 0.00 | 0.00 |

***: Values represent the means of three independent replicates**
